# Accuracy of Magnetic Resonance Imaging in Prenatal Diagnosis of Choledochal Cysts: A Single-Center Retrospective Analysis

**DOI:** 10.1155/2022/3268797

**Published:** 2022-09-26

**Authors:** Huiying Wu, Jinsheng Tian, Hehong Li, Hongsheng Liu, Yutao Liu, Lianwei Lu, Xiwen Chen, Xiaochun Zhang, Wenbiao Xu

**Affiliations:** Department of Radiology, Guangzhou Women and Children's Medical Center, Guangdong Provincial Clinical Research Center for Child Health, Guangzhou 510623, China

## Abstract

**Background:**

The purpose of this study is to evaluate the accuracy of prenatal MRI in diagnosing choledochal cysts (CDC), evaluate the sensitivity and specificity of MRI signs in the diagnosis of fetal CDC, and first compare the trend of size of CC between prenatal and postpartum.

**Methods:**

A total of 18 fetal who were diagnosed with CDCs through prenatal MRI were enrolled in the study. We summarized and analyzed the prenatal clinical data and prognosis information of prenatal and postpartum surgery, then compared the sensitivity, specificity, and diagnostic accuracy of various signs of MRI and postpartum MRCP diagnosis of CC. Finally, we tried to compare the earliest prenatal detection of common bile duct cysts with the size of surgery, and calculated the growth rate of common bile duct cysts for the first time.

**Results:**

All 18 patients were delivered in our institution. Among these patients, 14 were confirmed with CDCs after postpartum surgery, two patients had CDCs that disappeared, and two patients were confirmed with cystic biliary atresia (CBA) through the Kasai operation. Furthermore, 13 patients with CDCs and two patients with CBA underwent MRCP before the operation, and one patient with CDCs ruptured at birth and underwent ultrasound diagnosis. The sensitivity and diagnostic compliance of prenatal MRI signs for the location were higher when compared to postnatal MRCP (100% vs. 76.9% and 83.3% vs. 66.7%): the cyst was located at the porta hepatis, which was higher than the lowest edge of the liver, and parallel to the hepatoduodenal ligament.

**Conclusion:**

Prenatal MRI is higher than that of US for diagnosing CDCs, specifically in identifying the location of the cyst and confirming the origin of the cyst. The length, width, and size of the CDC become slightly bigger in our study.

## 1. Background

Choledochal cysts (CDC), also known as “congenital biliary dilatation,” account for approximately 1% of all benign biliary tract diseases. The incidence of CDCs in the Asian population is 1 : 1,000 and is lower than that in Western countries (1 : 100,000 to 1 : 150,000) [1–5]. CDCs are usually a surgical problem in infancy or childhood. The study conducted by Diao et al. [6] provided convincing data through a prospective randomized trial of a fairly large number of neonates, and it was revealed that early surgical intervention is advocated to prevent the deterioration of liver function, coagulopathy, and other severe complications.

Fortunately, the diagnosis of CDCs has gradually turned from after birth to prenatal due to the accuracy of prenatal ultrasounds (US). In the prenatal US, fetal CDCs have been described as follows: anechoic cysts located in the right upper quadrant of the abdomen, the subhepatic, or the region of the porta hepatis, with no septum, and these are connected to the gallbladder or hepatic ducts [7]. The latter two have always been considered as decisive signs in the prenatal diagnosis [8], but these could not be observed in all prenatal CDC cases [9], and the accuracy for the prenatal diagnosis of CDCs is only 7.7% [10]. Fetal CDCs may be misdiagnosed as origin-uncertain abdominal cysts, duodenal atresia, hepatic cysts, ovarian cysts, mesenteric cysts, renal abnormalities, normal gallbladder, or situs inversus [11].

At the same time, fast magnetic resonance imaging (MRI) was considered a useful tool for evaluating fetal abdominal disorders [12]. MacKenzie et al. [13] previously reported the prenatal ultrafast MRI finding of a choledochal cyst in a fetus at 16 weeks of gestation. Liu and Shih [14] considered that fetal MRI could provide more precise spatial images, when compared to US, for the identification of CDCs, and provide more information in the differential diagnosis. However, to the authors' knowledge, the ability of fetal MRIs to detect CDCs remains unclear.

The aim of the present study was to use prenatal MRI to define the features of CDCs and analyze the relationship among the prenatal MRI findings, the postnatal MRCP, and outcomes. This study also aimed to evaluate the key MRI findings of fetal CDCs in order to improve the diagnosis accuracy of prenatal MRI.

## 2. Methods

### 2.1. Clinical Data

**Figure 1:**
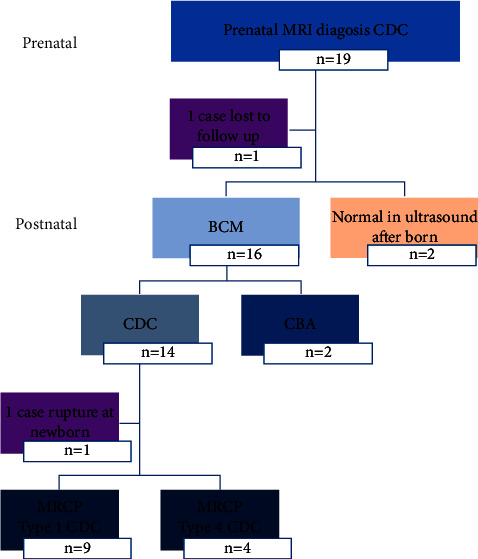
Study design and case descriptions.

Guangzhou Women and Children's Medical Center Ethics Committee approved (NO 2015090860) this study. The adverse effects of these MRI examinations were fully explained to each patient, and all patients in the present study provided signed informed consent. Fetal MRI was usually limited to cases in which the US results were indeterminate. In consideration of the importance of follow-up and the special advantages of US, serial US and postnatal magnetic resonance cholangiopancreatography (MRCP) examinations were necessary to observe the disease progress after the diagnosis of CDCs by prenatal MRI. Between January 2009 and September 2019, 26 patients were diagnosed with CDCs by fetal MRI after ultrasounds found fetal abdominal cysts. The inclusion criteria were as follows: (1) the prenatal care, delivery, and postpartum treatment were carried out in our institution with the appropriate perinatal counseling and management; (2) all prenatal and postnatal medical records were complete; (3) all pregnant women in the study were informed about the safety of the examinations and the possible complications of the operation and provided signed informed consent. The exclusion criteria were as follows: (1) in addition to CDCs, any cases of severe malformations, such as severe fetal cardiac malformation; (2) pregnant women or fetuses who were not available for follow-up; and (3) parents who refused the study of their babies. Those cases which could not be followed up (7 cases) and those without postpartum imaging data were excluded (1 case). Among these 18 patients, 2 (2/18, 11.1%) patients' postnatal US revealed that the cyst was not any more visible. 2 (2/18, 11.1%) patients were confirmed with cystic biliary atresia (CBA) through intraoperative cholangiography, and 14 (14/18, 77.8%) were confirmed with CDCs after postpartum surgery (Figure 1).

The clinical charts were reviewed. Clinical parameters, including the gestational age on the diagnosis of the CDC (prenatal MRI) and birth, birth weight, mode of delivery, date of MRCP after birth and date of surgery, symptoms at the time of operation, surgical procedures performed, and postoperative outcome data were collected and analyzed. The follow-up duration was measured from the definitive surgery to the last clinical assessment.

### 2.2. Fetal MRI and Postnatal MRCP Examination

As a preparation for the fetal MRI, the pregnant women were informed not to eat or drink three hours before the examination. An attempt was made to choose a time when the fetus was not active for the MRI examination. All fetal MRI and postnatal MRCP examinations were performed on an Achieva 1.5 T MRI scanner (Philips Healthcare, Netherlands). The fetal MRI was performed with the following scanning sequences: (1) T1-weighted imaging (WI): repetition time (TR) was 159 ms, echo time (TE) was 5.3 ms, the field of view (FOV) was 405 × 364 × 90 mm, and the matrix was 232 × 165; (2) T2-WI: TR was 15,000 ms, TE was 120 ms, the FOV was 400 × 400 × 75 mm, and the matrix was 248 × 217.

As a preparation for the postnatal MRCP, all children fasted for 4–6 hours and were given 0.5 ml/kg of 10% chloral hydrate before the examination. The MRCP was performed when the children were asleep, using a 5-channel phased-array heart parallel acquisition coil. The MRCP was performed with the following scanning sequences (1) axial fast gradient echo (FFE) T1WI: TR was 10 ms and TE was 4.6 ms; (2) single-shot TSE T2-WI: TR was 375.8–661.6 ms, and TE was 80 ms; (3) coronal 2D-balance fast field echo (2D-BFFE) T2-WI: TR was 4.0–7.1 ms, TE was 1.99–3.60 ms, the thickness was 5 mm, and there was no layer spacing; (4) 3D-TSE T2-WI: TR was 1,165.7–1,272.0 ms, TE was 650 ms, layer thickness was 0.8 mm, there was no layer spacing, and the matrix was 200 × 200.

**Figure 2:**
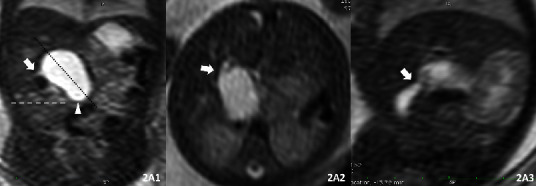
Signs of the CDC^*∗*^ (Case 11, G29 + W). Sign A: the cyst was located at the porta hepatis. Sign B: the end of the cyst is higher than the lowest edge of the liver (white dotted line). Sign C: parallel to the hepatoduodenal ligament (dark dotted line). Sign E: the cyst was connected to the gallbladder/duct of gallbladder (white arrow). Tapper sign (white triangle). ^*∗*^CDC: choledochal cysts.

**Figure 3:**
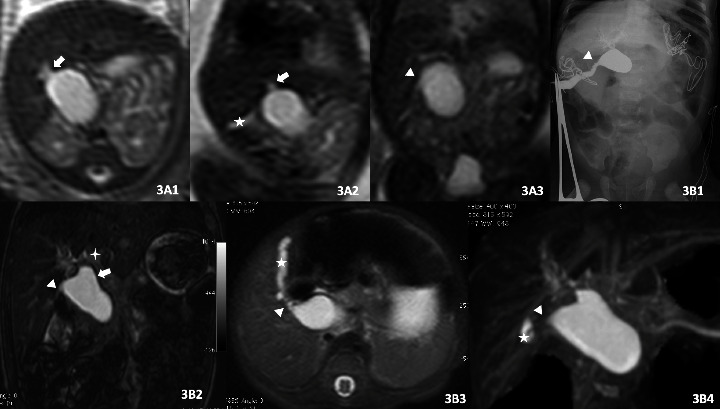
Case 8, G23 with CBA^*∗*^. A1–3: fetal MRI. Coronal (4A2–4A3) showed the cyst located in the hepatic hilar, parallel to the hepatoduodenal ligament, higher than the lower edge of the liver, and connected to the hepatic duct (white arrow) and gallbladder (white star)/duct (white triangle). Axial (4A1) showed the intrahepatic bile duct (white arrow) slight dilation. 16D postnatal MRCP^*∗*^ (B2–B4) showed that the size of cysts did not change much. The hepatic ducts were slightly dilated (white arrow), the gallbladder (white star) was stiff and caterpillar-like. Coronary T2WI (B2) showed mild edema around the portal vein (white stars). Preoperative cholangiography (B1) confirmed cystic biliary atresia. ^*∗*^CBA: cystic biliary atresia. ^*∗*^MRCP: magnetic resonance cholangiopancreatography. ^*∗*^T2WI: T2 weight imaging.

The MRI and MRCP results were, respectively, reanalyzed by two radiologists, who have six and ten years, respectively, of experience in analyzing fetal and pediatric MRI examinations, and were blinded to the diagnosis and postnatal outcomes. The routine prenatal MRI imaging focused on the following signs in these subjects: Sign A: the cyst was located at the porta hepatis (Figure 2A1); Sign B: the end of the cyst was higher than the lowest edge of the liver (Figure 3); Sign C: parallel to the hepatoduodenal ligament (Figure 2A1); Sign D: the cyst was connected to the hepatic ducts (Figure 3); Sign E: the cyst was connected to the gallbladder or the duct of the gallbladder (Figures 2A1–2A3); Sign F: dilated intrahepatic bile ducts (left and right hepatic ducts, Figure 3). Tapered sign: the coronal T2-weighed image reveals the CDC tapered ends [15] (Figure 2A1). All prenatal MRI and postnatal MRCP were divided into types I–V according to Todani classifications of CDCs [16]. The length and width of the CDC and gallbladder were measured twice in all cases, the average value was taken, and the area was calculated. If the tworadiologists did not reach an agreement, the results were determined after reaching a consensus through discussions.

### 2.3. Statistical Analysis

The statistical analysis was performed using SPSS 23.00 (Statistical Analysis System, Chicago, IL, USA). A significance was achieved at the 0.05 level. Only significant *P*-values are indicated.

A statistical diagnostic test was used to calculate the sensitivity, specificity, and diagnostic compliance of each diagnosis by prenatal MRI and postnatal MRCP.

After the diagnosis of the CDC, the investigators attempted to estimate the trend size of the cyst and gallbladder in two MRI examinations. The *k*-*s* normality test was used to test the continuous variables: the length, width, and area of the CDC and gallbladder. The continuous variables data were presented as mean ± standard deviation (SD). If the data followed the normal distribution, the differences were compared by paired Student's *t*-test. If the data did not follow the normal distribution, the independent sample rank-sum test was used to compare the differences.

## 3. Results

### 3.1. Clinical Data

The 18 fetuses were diagnosed with CDCs by MRI, with a mean gestational age of 28.2 ± 5.2 weeks (21–38 weeks). The prenatal screening of trisomy 21 and 18 in all cases revealed a low risk. All 18 fetuses were delivered in our institution (Guangzhou Women and Children's Medical Center). The clinical data are listed in Table 1.

There was a total of 14 cases of CDC, with a mean gestational age on diagnosis (fetal MRI) and at birth of 28.6 ± 5.5 weeks (21–38 weeks) and 38.9 ± 1.1 weeks (37–40 weeks), respectively. Six (42.9%) cases were detected in the second trimester, and eight (57.1%) were detected in the third trimester. There were six (42.9%) cases of routine delivery and eight (57.1%) of cesarean sections. The mean newborn weight was 3,439.9 ± 278.6 g, which ranged within 2,760–3,800 g. Among the newborns, six (42.9%) were male, and eight (57.1%) were female. The mean age of children with CDCs who underwent MRCP was 94.1 ± 147.5 days, which ranged within 3–480 days, while one case where the CDC ruptured after birth only underwent US.

Ten (71.4%) cases exhibited symptoms before surgery, including seven (50.0%) cases of jaundice, three (21.4%) of vomiting and abdominal distension, and two (14.3%) of abdominal mass. All 14 patients with CDCs underwent surgery at the mean age of 105.6 ± 149.4 days (3–480 days). Among these cases, 11 (78.6%) cases were confirmed as type I CDC, and three (21.4%) were confirmed as type IV CDC. Six (42.9%) cases underwent laparotomy, and eight (57.1%) underwent open surgery of Roux-en-Y biliary reconstruction. The average duration of MRCP and operation was 19.4 ± 34.1 days (0–120 days). The follow-up period varied within 9–84 months after birth, with an average period of 48.9 ± 26.2 months.

### 3.2. Prenatal MRI Findings of CDC

The diagnostic accuracy of CDCs was 77.8% (14/18) in the prenatal MRI and 86.7% (13/15) in the postnatal MRCP. The sensitivity, specificity, and diagnostic accuracy of each sign are calculated and summed up in Tables 2 and 3.

1. Location (Signs A and C), size, and shape: Sign B was mainly determined by the size of the CDC. The maximum diameter of 85.7% (12/14) of the CDC was less than 3.0 cm, and 92.9% (13/14) of the CDC had an edge higher than the lowest edge of the liver. The shape of the CDC was oval or round, which was similar to the other fetal cyst in the abdomen.
2. Biliary origin (Sign D–F): Sign D had the highest sensitivity (100%) and diagnostic accuracy (88.9%) among all signs in the prenatal diagnosis. In one case, the cyst was connected to the gallbladder, but this was subsequently confirmed to be the back fold of the gallbladder (Figures 2A1–2A2). In the other case, the cyst appeared to be connected to the gallbladder, which was obviously compressed and flattened (Figures 2B1–2B2), and the cyst could not be found after birth. Furthermore, 100% (14/14) of the CDC did not reveal the dilation of the intrahepatic bile ducts, while 28.6% (4/14) presented with dilations before the operation.
3. Tapered sign: this has the lowest sensitivity among all the prenatal MRI and postnatal MRCP (21.4% and 7.7%, respectively) in the CDC diagnosis.
4. Characteristics of the cyst: all these signs were negative in all the prenatal MRI.
5. Type of Todann: in the prenatal MRI, 17 (94.4%) cases were diagnosed as type I CDC, and 1 (5.6%) was diagnosed as type IV CDC.

### 3.3. Dynamic Trend of Length, Width, and Size of the CDC and Gallbladder

The size of cysts and gallbladder was calculated according to the prenatal MRI and postnatal MRCP results. The length, width, and size of the CDC and gallbladder followed the normal distribution, and a paired Student's *t*-test was conducted.

1. The length, width, and size of the CDC: the mean length, width, and size of the CDC were 2.3 ± 0.8 cm, 1.7 ± 0.4 cm, and 0.7 ± 0.3 cm^2^, respectively, at the prenatal MRI, and 5.1 ± 2.0 cm, 3.5 ± 1.8 cm, and 20.6 ± 18.6 cm^2^, respectively, at the postnatal MRCP. The differences in length, width, and size of the CDC at the prenatal MRI and postnatal MRCP were statistically significant (*P* ≤ 0.001). The length, width, and size of the CDC were larger in cases that underwent postnatal MRCP when compared to cases that underwent prenatal MRI. In order to eliminate the effect of these intervals, the area of the cyst was divided by the interval time (the interval between two MRI examinations). The mean of the growth area of the CDC was 0.2 ± 0.4 cm^2^.
2. Size of the gallbladder: the mean of the length, width, and size of the gallbladder was 1.0 ± 0.3 cm, 0.7 ± 0.2 cm, and 0.7 ± 0.3 cm^2^, respectively, at the prenatal MRI, and 1.4 ± 0.7 cm, 0.8 ± 0.3 cm, and 1.2 ± 0.9 cm^2^, respectively, at the postnatal MRCP. The differences in length, width, and size of the gallbladder in prenatal MRI and postnatal MRCP were not statistically significant (*P* > 0.005). This infers that the size of the gallbladder did not change over time.

## 4. Discussion

### 4.1. Clinical

Five types of CDCs have been described according to Todani classifications. Type I cysts constitute 80%–90% of CDCs [17], and type IV cysts are more common in adults [18] than in children. It is noteworthy that all fetal CDCs were type I in the prenatal MRI performed for the present study, and three cases of type I CDCs changed to type IV after the operation. The dynamic change in the size of the CDC increases the possibility that although these lesions may be congenital, they may progress over time, for example, type IV CDCs may develop from type I CDCs. This may also be due to the large thickness layer of the prenatal MRI, which may cause the dilated intrahepatic bile ducts to appear unclear. Only one case of “type IV CDC” in the prenatal MRI was confirmed to be CBA. It remains unknown whether the dilatation of the intrahepatic bile duct is secondary to the dilatation of the extrahepatic bile duct.

Spontaneous perforation of CDCs is rare in children [19, 20]. The cause of the perforation remains debatable. Some researchers have proposed an increase in intraluminal pressure within the CDC [21]. A newborn with CDCs, who presented with perforation, required emergency surgery with CDC drainage in the present study. However, the cyst was not large (1.5 × 1.9 cm) in the prenatal MRI at G34 W, indicating that the size of the CDC might not be the main cause of the rupture of the CDC. The mode of delivery and weight of the baby may also be possible risks that contribute to the perforation of CDCs. Since the possible cause of a CDC rupture is currently unknown, the investigators consider that children with suspected CDCs during the fetal period need to undergo an early US review after delivery.

### 4.2. MRI Finding of Fetal CDC

The present “gold standard” for the diagnostics and stages of CDCs is MRCP [22]. However, MRCP shows the limited capacity to detect the associated ductal anomalies or small choledochocele [23], and few studies have focused on CDC's diagnosis at the fetal stage. The prenatal MRI diagnostic accuracy was 77.8% in the present study, and this was much higher than that of prenatal US (7.7%) [10]. Prenatal MRI is performed when ultrasound finds abnormalities or suspects CDCs, so the diagnosis accuracy of prenatal MRI will be higher than that of ultrasound. Radiologists will look for other signs based on what the ultrasound has found.

The prenatal MRI could indicate the location and source of all CDCs, which were located in the hepatic hilum and originated from bile ducts. The maximum diameter of fetal CDC may be an interesting sign to identify CDCs from other abdomen cysts in the prenatal stage. The maximum diameter of CDCs was 4.6 cm, and 85.6% of these cases were <3 cm within 21–38 weeks of gestation (mean: 28 weeks of gestation). As the size of the CDC increased, the specificity of these three signs decreased to 76.9%, 38.4%, and 23.0%, respectively, in the postnatal MRCP.

The incidence that shows that the CDC communicates with the intrahepatic bile duct or with the bile duct and gallbladder in the prenatal MRI was the same as that in the postnatal MRCP. However, this was also the most difficult MRI sign to confirm in the prenatal diagnosis. The thickness of the fetal MRI was 4–6 mm, and the connection of the cyst and bile duct and gallbladder could only be revealed in one to two slices in each sequence. In the situation, it is hard for the radiologist to confirm the real connection and to determine whether there was a pancreaticobiliary maljunction of fetal CDCs. A folder gallbladder and large cyst would compress the gallbladder, leading to a misdiagnosis, which is similar to Case 5.

Wong et al. [15] reported that the “tapered sign” was one of the characteristics that could be inferred as a CDC. In the present study, the sensitivity of tapered signs in both the prenatal and postnatal MRI was too low (21.4% and 7.7%, respectively). Furthermore, the tapered sign appeared to be less obvious with the increase in the size of the CDC, and the decrease in its sensitivity in MRCP. The pedicle of the cyst may also show the tapered sign. Similar to case 10, a cyst presented in the third trimester and showed the tapered sign. Given that there were no reports of the disappearance of the CDC, the “tapered sign” may be caused by a huge pedicled ovarian cyst located at the junction of the liver and the gallbladder. There were few “internal characteristic signs” of CDCs in the prenatal MRI. All cysts are oval or round, thin-walled, and unilateral. In the present study, the wall of the prenatal CDC could not be distinguished, the presence of a septal in the CDC could not be found, and no bile duct stones could be detected.

At present, it is difficult to differentiate between CDCs and cystic biliary atresia (CBA), as both are cystic dilatations of the biliary tree [24]. CBA is a special variant of biliary atresia (BA), which accounts for 10% of BA [25] and present in 22% of fetal cystic dilatations of the biliary tree [26]. CBA was present in 12.5% of fetuses diagnosed with CDCs in the present study. All MRI signs mentioned above, in theory, can only confirm that the cyst originated from the biliary tract. The detection of an existing gallbladder issue was considered another clue to identify CBA from CDCs [27]. In our study, the entire gallbladder was analyzed, and the dynamic postnatal size of the gallbladder was the same as its prenatal size. But the size of the CDC appeared to be growing gradually, and the size of two cases of CBA was relatively the same in the present study.

For patients who have no jaundice, and those who do not have an increase in cyst size, elective excision at a later age is advocated [28]. However, in view of these slow changes in the size of CDCs, distinguishing CDCs from CBA based on the changes in cyst size appears to be difficult for infants under two months. We recommend that infants younger than two months should have a biliary angiography to rule out CBA when the size of the cyst does not significantly change, and the diagnosis is unclear. In the present study, it was only observed that the size of the CDC slowly grew. The growth rate of the cyst in the fetus and after birth may provide a basis for a more detailed assessment of CDCs and the differentiation of CBA. CBA is also the main reason why the specificity and diagnosis compliance of the fetal MRI and MRCP were not high in the present study.

### 4.3. Differential Diagnosis

Fetal abdominal cystic lesions are relatively common and have an incidence of 1/1,000 [11]. The abnormal cystic structures mainly originate from either the gastrointestinal tract or the genitourinary tract [29]. The location, morphology, tension of the cyst, the wall thickness, motility, and dynamic changes are the main identification points for fetal abdominal cysts (Table 4).

### 4.4. Limitations

There were several limitations to the present study. First, the sample size was too small to make broad generalizations. Second, not all fetal abdomen cyst patients with fetal MRI were chosen for comparison, and the other cyst outcomes were not compared. Third, the present study was a retrospective study. These diagnostic criteria should be used for prospective studies.

## 5. Conclusions

In conclusion, the diagnostic accuracy of prenatal MRI for CDCs is higher than that of US. Fetal MRI is better than ultrasound in showing the cyst position and relationship with the biliary tract. Of all the signs, the cyst is the highest sensitivity and specificity above the lower edge of the liver and connected to the intrahepatic bile duct. For the first time, we evaluated the daily growth rate of common bile duct cysts from fetal to preoperative period, and we found that the length, width, and size of the CDC may become larger over time.

## Figures and Tables

**Table 1:** Clinical data and postnatal outcomes of 18 fetus with CDC by MRI diagnosis.

No	Gender	GA with MRI	GA at birth	Weight at birth (g)	Mode of delivery	Symptom	Age of operation (d)	Type of CDC	Way of operation	Diagnosis	Follow-up (months)
1	*M*	G22+	G38+	3650	Cesarean section	Jaundice	5	Type 1	Roux-en-Y (laparoscope)	CDC	16 m
2	*M*	G21+	G37+	3448	Cesarean section	Jaundice	30	Type 1	Roux-en-Y (laparoscope)	CDC	15+ m
3	*M*	G26+	G39+	3800	Vagina	Jaundice	30	Type 1	Roux-en-Y (laparoscope)	CDC	9+ m
4	*F*	G25+	G40+	3800	Cesarean section	NA	90	Type 1	Roux-en-Y (laparoscope)	CDC	14+ m
5	*M*	G24+	G37+	3160	Vagina	NA	NA	NA	NA	Normal	27 m
6	*F*	G29+	G38+	3270	Cesarean section	Jaundice	60	NA	Kasai operation (OS)	BA	28+ m
7	*F*	G24+	G40+	3670	Vagina	NA	60	Type 4	Roux-en-Y(Laparoscope)	CDC	39 m
8	*F*	G23+	G39+	3440	Cesarean section	Jaundice	60	NA	Kasai operation (OS)	BA	36 m
9	*F*	G21+	G40	3350	Cesarean section	NA	35	Type 1	Roux-en-Y (laparoscope)	CDC	48 m
10	*F*	G32+	G40	3180	Vagina	NA	NA	NA	NA	Normal	36 m
11	*F*	G29+	G38+	2760	Cesarean section	Abdominal mass	30	Type 1	Roux-en-Y (laparoscope)	CDC	53 m
12	*F*	G29+	G37+	3260	Cesarean section	Vomiting, abdominal mass	210	Type 4	Roux-en-Y (laparoscope)	CDC	52 m
13	*M*	G31+	G38+	3690	Cesarean section	NA	26	Type 1	Roux-en-Y (OS)	CDC	67 m
14	*F*	G34+	G39+	3280	Vagina	Vomiting, abdominal distension	26	Type 1	Roux-en-Y (OS)	CDC rupture	71 m
15	*F*	G38+	G39	3450	Cesarean section	Jaundice, vomiting, abdominal distension	300	Type 4	Roux-en-Y (OS)	CDC	65 m
16	*M*	G35+	G39	3380	Vagina	Jaundice, vomiting, abdominal distension	7	Type 1	Roux-en-Y (OS)	CDC	75 m
17	*F*	G32	G40	3420	Vagina	Jaundice	3	Type 1	Roux-en-Y (OS)	CDC	84 m
18	*M*	G33	G40	3200	Vagina	Jaundice	480	Type 1	Roux-en-Y (OS)	CDC	77 m

*Note.* Roux-en-Y hepaticojejunostomy; open surgery: OS.

**Table 2:** MRI diagnosis results of 18 fetus with CDC.

No	Gender	Prenatal MRI											Postnatal MRCP											
		Time of MRI	Cyst (long × width) cm	GB (long × width) cm	Sign A	Sign B	Sign C	Sign D	Sign E	Sign F	Tapered sign	Type of todani	Age of MRCP	Cyst (long × width) cm	GB (long × width) cm	Sign A	Sign B	Sign C	Sign D	Sign E	Sign F	Tapered sign	Type of todani	Diagnosis results
1	*M*	G22+	2.5 × 3.3	1.0 × 0.7	+	−	+	+	+	−	−	Type 1	7 d	5.3 × 6.6	1.1 × 0.7	+	−	−	+	+	−	−	Type 1	CDC
2	*M*	G21+	1.6 × 1.8	1.0 × 0.8	+	+	+	+	+	−	+	Type 1	30 d	3.2 × 5.4	1.1 × 1.0	+	+	−	+	+	+	−	Type 4	CDC
3	*M*	G26+	1.3 × 1.9	1.6 × 0.9	+	+	+	+	+	−	−	Type 1	30 d	1.5 × 4.2	1.5 × 0.8	+	−	+	+	+	−	−	Type 1	CDC
4	*F*	G25+	1.0 × 1.8	0.6 × 0.3	+	+	+	+	+	−	−	Type 1	90 d	2.3 × 4.1	1.3 × 0.9	+	−	+	+	+	−	−	Type 1	CDC
5	*M*	G24+	0.7 × 2.5	0.9 × 0.6	+	−	−	+	+	−	−	Type 1	NA	NA	NA	NA	NA	NA	NA	NA	NA	NA	NA	Normal
6	*F*	G29+	0.9 × 1.1	2.7 × 0.3	+	+	+	−	−	−	−	Type 1	35 d	0.8 × 1.0	NA	+	+	+	+	+	−	−	NA	BA
7	*F*	G24+	1.6 × 2.1	0.9 × 0.7	+	+	+	+	+	−	−	Type 1	6 d	2.1 × 5.5	1.2 × 0.9	−	−	−	+	+	+	−	Type 4	CDC
8	*F*	G23+	1.7 × 2.6	0.8 × 0.6	+	+	+	+	+	+	−	Type 4	16 d	3 × 1.5	0.9 × 0.5	+	+	+	+	+	+	+	NA	BA
9	*F*	G21+	1.7 × 1.9	0.7 × 0.7	+	+	+	+	+	−	−	Type 1	30 d	1.8 × 2.2	0.8 × 0.7	+	+	+	+	+	−	−	Type 1	CDC
10	*F*	G32+	4.1 × 4.8	2.6 × 1.5	−	−	+	−	+	−	+	Type 1	NA	NA	NA	NA	NA	NA	NA	NA	NA	NA	NA	Normal
11	*F*	G29+	2.1 × 2.2	0.7 × 0.6	+	+	+	+	+	−	−	Type 1	4 d	3.8 × 6.0	1.6 × 0.6	−	−	−	+	+	−	−	Type 1	CDC
12	*F*	G29+	1.4 × 2.2	0.9 × 0.9	+	+	+	+	+	−	+	Type 1	210 d	7.0 × 8.5	2.1 × 1.0	+	−	−	+	+	+	−	Type 4	CDC
13	*M*	G31+	1.5 × 1.5	1.3 × 0.8	+	+	+	+	+	−	−	Type 1	26 d	2.1 × 3.4	1.0 × 0.7	+	+	+	+	+	−	−	Type 1	CDC
14	*F*	G34+	1.5 × 1.9	1.4 × 0.8	+	+	+	+	+	−	−	Type 1	NA	2.3 × 1.5^*∗*^	0.6 × 0.7^*∗*^	NA	NA	NA	NA	NA	NA	NA	Type 1	CDC^*∗*^
15	*F*	G38+	2.3 × 2.3	0.9 × 0.6	+	+	+	+	+	−	+	Type 1	300 d	5.3 × 5.0	3.5 × 1.0	+	−	−	+	+	+	−	Type 4	CDC
16	*M*	G35+	2.4 × 4.6	0.9 × 0.6	+	−	+	+	+	−	−	Type 1	7 d	6.4 × 9.2	0.8 × 0.5	−	−	−	+	+	−	−	Type 1	CDC
17	*F*	G32	1.6 × 2.7	0.8 × 0.6	+	+	+	+	+	−	−	Type 1	3 d	3.2 × 2.1	1.0 × 0.7	+	+	+	+	+	−	+	Type 1	CDC
18	*M*	G33	2.3 × 1.5	0.7 × 0.6	+	+	+	+	+	−	−	Type 1	480 d	3.3 × 2.6	1.6 × 1.4	+	+	+	+	+	−	−	Type 1	CDC

*Note.* ^*∗*^the number came from the ultrasound report when the CDC rupture; Sign A: the cyst was located at the porta hepatis; Sign B: the end of the cyst is higher than the lowest edge of the liver; Sign C: parallel to the hepatoduodenal ligament; Sign D: the cyst was connected to the hepatic ducts; Sign E: the cyst was connected to the GB/duct of GB; Sign F: dilated intrahepatic bile ducts (left and right hepatic ducts).

**Table 3:** Diagnostic test results of prenatal MR single signs in suspicious CDC cases.

MRI sign of CDC	Prenatal MRI			Postnatal		
	Sensitivity	Specificity	Diagnostic compliance	Sensitivity	Specificity	Diagnostic compliance
Sign A: the cyst was located at the porta hepatis	100% (14/14)	25% (1/4)	83.3% (15/18)	76.9% (10/13)	−(0/2)	66.7% (10/15)
Sign B: the end of the cyst is higher than the lowest edge of the liver	92.9% (13/14)	50% (2/4)	83.3% (15/18)	38.4% (5/13)	−(0/2)	33.3% (5/15)
Sign C: parallel to the hepatoduodenal ligament	100% (14/14)	25% (1/4)	83.3% (16/18)	23.0% (6/13)	−(0/2)	40% (6/15)
Sign D: the cyst was connected to the hepatic ducts	100% (14/14)	50% (2/4)	88.9% (16/18)	100% (13/13)	−(0/2)	86.7% (13/15)
Sign E: the cyst was connected to the GB/duct of GB	100% (14/14)	25% (1/4)	83.3% (15/18)	100% (13/13)	−(0/2)	86.7% (13/15)
Sign F: dilated intrahepatic bile ducts (left and right hepatic ducts)	−(0/14)	75% (3/4)	16.7% (3/18)	30.1% (4/13)	50% (1/2)	33.3% (5/15)
Tapered sign: coronal T2-weighed image showed the choledochal cyst's tapered ends	21.4% (3/14)	75% (3/4)	33.3% (6/18)	7.7% (1/13)	50% (1/2)	13.3% (2/15)

**Table 4:** Differential of fetal abdominal cyst.

	Location	Morphology	Tension	Wall thickness	Motility	Dynamic changes	Features
Ovarian cysts	Lower abdomen/pelvis and at one side of the bladder	Round-shaped	+	Thin	Slightly	Disappear after birth or continue to grow and present with torsion following birth.	The most common cyst in female fetal

CDC (CBA)	Located at the porta hepatis	Oval/round-shaped	+	Thin	Fixed	Continue to grow and present with rupture following birth.	Female: male = 3–4 : 1

Intestinal duplication	In the middle of the abdomen and close to the intestine	Oval-shaped	+	Thick	Fixed	Remain the same or slightly increase following birth.	Intestinal wall-like structure

Mesenteric cyst	Near the mesenteric edge of the small intestine.	Unilocular or multilocular	−	Thin	Fixed	Remain the same or slightly increase following birth.	—

## Data Availability

The datasets used and/or analyzed during the current study are available from the corresponding author on reasonable request.

## Abbreviations

MRI: Magnetic resonance imaging
MRCP: Magnetic resonance cholangiography
ERCP: Endoscopic retrograde cholangiopancreatography
CDC: Choledochal cyst
CBA: Cystic biliary atresia
BA: Biliary atresia
US: Ultrasound
BCM: Biliary cystic malformation
FOV: Field of view
TR: Repetition time
TE: Echo time
GB: Gallbladder.

## Additional Points

The length, width, and size of the CDC may become larger over time. The prenatal MRI would directly affect the accuracy of the diagnosis and the proper treatment of the CDC. Performing an accurate antenatal diagnosis of CDCs can facilitate a definitive treatment.
